# Modified minimally invasive approach and intra-osseous portal for three-part proximal humeral fractures: a comparative study

**DOI:** 10.1186/s13018-017-0701-1

**Published:** 2018-02-01

**Authors:** Zhuo Zhang, Gongzi Zhang, Ye Peng, Xiang Wang, Hui Guo, Wei Zhang, Peifu Tang, Lihai Zhang

**Affiliations:** Orthopedic Department, Chinese PLA General Hospital, Fuxing Road, 28, Beijing, 100853 People’s Republic of China

**Keywords:** Three-part proximal humeral fracture, Minimally invasive, Deltopectoral, Approach, Locked plating

## Abstract

**Background:**

Proximal humeral fracture is a common fracture. Different approaches have been utilized in the surgical intervention of three-part fractures. Our study is to evaluate the clinical outcomes and effectiveness of a modified anterolateral approach and intra-osseous portal in minimally invasive treatment for three-part proximal humeral fractures in comparison to the traditional deltopectoral approach.

**Methods:**

From March 2015 to September 2016, 13 patients with three-part proximal humeral fractures were treated with internal fixation through the modified anterolateral minimally invasive approach (MIPO). These cases were compared to 20 additional cases using the deltopectoral approach (DP). Clinical and radiographic evaluations were performed, including the constant score (CS) and range of motion in abduction, flexion/extension and external/internal rotation. Complications were recorded as well.

**Results:**

All patients were followed up for a mean time of 12.12 ± 4.01 months. At the latest follow-up, no significant differences (*p* < 0.05) were observed in terms of length of stay, range of motion for abduction, flexion or internal/external rotation of the shoulder, Constant score or visual analog scors (VAS) for pain. Elbow flexion (142.31 ± 8.32 vs. 123.00 ± 10.18), posterior shoulder extension (41.92 ± 5.22 vs. 35.50 ± 5.83) and postoperative VAS (4.38 ± 1.04 vs. 6.15 ± 0.99) were significantly better in the MIPO group than in the DP group (*p* < 0.05). No significant differences were detected in the radiographic evaluation, and complications including axillary nerve injury were not present.

**Conclusion:**

The use of the modified anterolateral approach and intra-osseous portal is safe and effective for minimally invasive reduction and plating treatment for three-part proximal humeral fractures.

## Background

Proximal humeral fracture (PHF) is a common fracture, accounting for 5% of total fractures and 39.7% of humeral fractures in the Chinese population [1]. Fracture of the proximal humerus is the third most common fracture among the geriatric population, following fractures of the distal radius and proximal femur. As the size of the geriatric population is rapidly increasing, an increased incidence of PHF is expected [2].

Proximal humeral fractures have been treated in a variety of ways, ranging from conservative to operative treatments. Considering the poor bone quality of the proximal humerus in aging patients, locking plate fixation remains the standard for surgical treatment. Different approaches for plating proximal humeral fractures have been described in the literature, including the classic deltopectoral approach and deltoid splitting approach. The classic deltopectoral approach has been used for years, especially for the open reduction and internal fixation (ORIF) of three- and four-part proximal humeral fractures. However, this approach provides limited access to the posterolateral aspect of the shoulder, and viewing the retracted greater tuberosity fragment in this area may be difficult. Moreover, the surgical exposure itself and overzealous dissection during plating may increase the risk of osteonecrosis due to injury of the ascending branch of the anterior circumflex humeral artery, which would lead to high rates of complication [3, 4]. The deltoid splitting approach was originally used for arthroplasty. In recent years, more attention has been paid to its utility in a plating procedure for proximal humeral fractures, through which the incidence of osteonecrosis may be reduced by minimizing interruption to constructs of the medial soft tissue hinge, although the hinge may be not be addressed in a deltopectoral DP approach. The minimally invasive approach utilizes either two incisions or one incision with a few distal percutaneous incisions. The proximal incision is part of the deltoid splitting approach, which is used for reduction and plate insertion, and the distal incision(s) are used for distal screw fixation. Typically, the MIPO approach has been utilized in the treatment of two-part fractures, and its use in three-part fractures is limited.

Although rarely reported, the deltoid splitting and minimal invasive approaches do put the axillary nerve at risk. Axillary neuropraxia is the most common nerve injury, and it resolves eventually in most cases, but it may be irreversible in some cases. All of the approaches discussed thus far require dissection of deltoid bursa during exposure and reduction, and limitations of range of motion may be observed due to postoperative adhesion and scars. We conducted this comparative study to evaluate the clinical outcomes and effectiveness of a novel approach, utilizing the modified minimal invasive approach and intra-osseous portal for reduction and plating of PHFs.

## Methods

### Demographic characteristics

From March 2015 to September 2016, 13 patients (7 males, 6 females) with three-part proximal humeral fractures were treated with plating through modified minimal invasive approach (MIPO). For comparison, 20 patients (7 males, 13 females) with the same fracture pattern who underwent ORIF through the deltopectoral approach were reviewed as well. Confirmation of diagnosis was made by a separate senior surgeon, who did not participate in the surgical procedures. The average age of patients was 66.15 ± 11.83 years old (range: 46 to 83) in the MIPO group and 61.55 ± 15.86 years old (range: 32 to 84) in the DP group.

### Implant

The Proximal Humerus Internal Locking System (PHILOS, Synthes, Oberdorf, Switzerland) was selected for internal fixation of all fractures.

### Surgical procedures

All patients were set in a beach-chair position with the shoulder of the surgical side free of motion. General anesthesia or regional block anesthesia was applied. All surgical procedures were performed by the same surgeon (L. Zhang), and the same surgical group participated.

### Deltopectoral approach

With the deltopectoral approach (DP), the long tendon of the biceps was identified, a procedure that we believe is essential for proper positioning of the plate outside of the bicipital groove. It is important to identify the long tendon of the biceps and to carefully avoid isolating it because the anterior circumflex artery enters the humeral head at the proximal end of the transition from the greater tuberosity to the intertubercular groove. The plate was placed anterolaterally according to its precontoured shape. A bone graft was inserted into the medial side of the proximal humerus for support.

### Modified MIPO approach

For the minimally invasive group, the MIPO technique was utilized. A K-wire was percutaneously inserted into the space underneath the acromion under fluoroscopy monitoring, while the tip of acromion was targeted. The axillary nerve travels from the posterior aspect of the humerus and traverses from a lateral to an anterior position nearly horizontally at the level of approximately 3–7 cm (average 5.7 cm along the lateral side and 5.1 cm along the anterior side of the arm) from the tip of acromion [5]. Projection of the axillary nerve was identified on the lateral aspect of the upper arm. The proximal longitudinal incision was made through the entry point, which extended 2–3 cm proximally and distally, respectively.

In a three-part fracture, there will be a fracture splitting either through the greater tuberosity or between the greater tuberosity and lesser tuberosity. After splitting the deltoid, the fracture of the lateral wall could be directly exposed. If the fracture cannot be directly exposed, the fracture is located between the greater and lesser tuberosities, and rotating the upper arm will help to visualize the fracture Tension-reducing rotator cuff sutures (Ethibond Excel 2/0 W4843, Ethicon USA, Cincinnati, USA) were hooked to the ligamentous inserts of each fragment for reduction control.

**Fig. 1:**
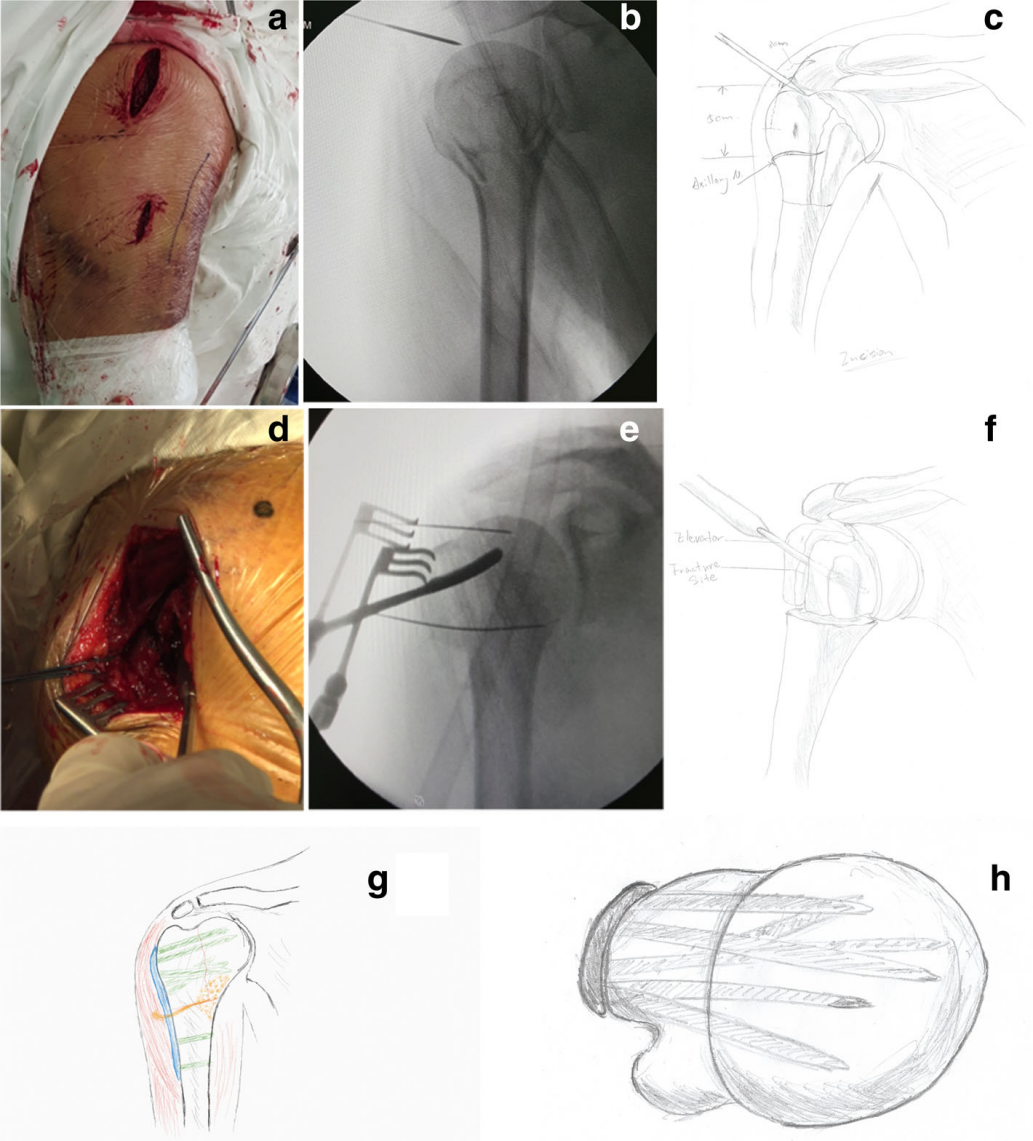
Surgical procedure of modified MIPO. **a** Incisions of MIPO procedure through deltoid splitting approach. **b,c** Targeting with a pin. **d, f** Reduction through fracture. **g, h** Plating and bone graft. Notice the position of axillary nerve. Both nerve and medial vascular structures are not disturbed

**Fig. 2:**
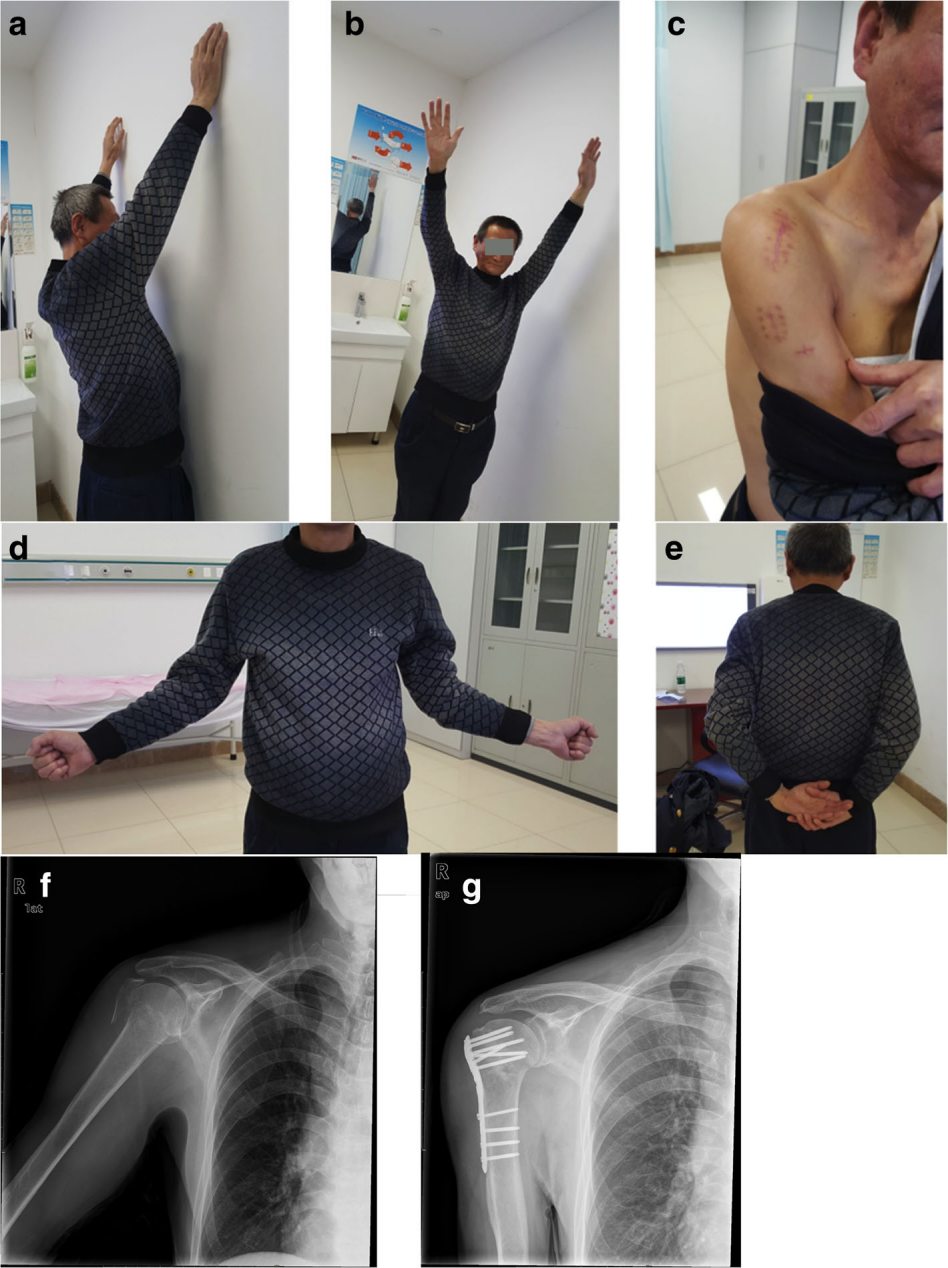
Clinical and functional outcome. **a**–**e** Function at the latest follow-up. **f** Preoperative X-ray. **g** Post-operative X-ray

A small elevator was then conducted from the bony portal inside the humeral head. Reduction of the fracture was achieved by pushing the fragments in each direction with the elevator, as well as restoring tension of the surrounding tendons and ligaments by tightening sutures on the inserts. After reduction of the cortex of the humeral head, a large cavity can be seen inside the humeral head, which requires a bone graft for more reliable fixation. Autografts or allografts were inserted through the portal to the medial head-neck junction area, resulting in abundantly reliable medial support to the proximal humerus, before closing the portal and providing temporary fixation. The distal fragment (diaphysis) was reduced in an indirect way by applying traction to the diaphysis of the humerus. After realignment of the distal fragment (via varus or posterior tilt of the humeral head), a precontoured locking plate (PHILOS) of proper length was inserted along the surface of the humerus, without dissecting the deltoid bursa, and temporally fixed by K-wires or guide wires. Proximal screws were fixed through the proximal incision, and distal screws were fixed percutaneously with the jig, following the principle of an MIPO procedure. Throughout this procedure, the risk area of the axillary nerve was bypassed (Fig. 1). The six multi-axial proximal locking screws provide enough grasping force, while the bone graft rather than a calcar screw supports the humeral head from postoperative migration (Fig. 2).

### Postoperative management

Cefuroxime (1.5 g) was given twice a day for 3 days for intravenous antibiotic prophylaxis. Analgesic agents were provided for pain control with 40 mg of parecoxib given intravenously twice a day for 3 days, and then patients were converted to oral protocols after the third postoperative day. All patients began rehabilitation as soon as the immediate postoperative radiographic examination was conducted. Rehabilitation included limited active and passive range of motion excises and strength recovery.

### Clinical and radiographic evaluation

The clinical assessment was performed by an individual examiner. The clinical results were documented by recording the constant score (CS) and range of motion in abduction, flexion/extension, and external/internal rotation. Complications, such as malreduction, neurovascular injury, fixation failure, bone absorption, malunion, or nonunion, were recorded as well.

For each patient, true anteroposterior and Y-view radiographs of the shoulder were obtained within 3 days postoperatively. The radiographs were analyzed for quality of fracture reduction and plate position by a blinded examiner. A minor varus or valgus head-shaft alignment of 110 to 150 degrees was considered to be acceptable, while alignment of < 110° or > 150° was rated as a malreduction, which is in agreement with published literature [3, 6, 7].

### Statistical analysis

All measurement data are provided as the mean values ± standard deviations, and differences between groups were evaluated by standard *t* tests. Descriptive data were recorded as ratios and evaluated by Chi-square tests. Statistical significance was defined as a value of *p* < 0.05. SPSS 22.0 statistical software for Macintosh (SPSS Inc., Chicago, IL) was used for data analysis.

## Results

All patients were followed up for a mean time of 12.12 ± 4.01 months (range 7 to 19; MIPO 12.46 ± 4.39, DP 11.90 ± 3.85, *p* = 0.320). Major complications, such as infection, implant failure, intraoperative fracture, or axillary nerve injury, were not seen in our study. Wound healing was satisfactory in all cases, and there were no delays of rehabilitation (Table 1).

**Table 1:** Clinical features and results

Patient No.	Group	Gender	Age (y/o)	Follow-up time (month)	Abduction (degree)	Extension (degree)	Flexion (degree)	I.R. (degree)	E.R. (degree)	C.S. (degree)	Elbow flexion (degree)	VAS 1	VAS 2	H-S angle (degree)	LOS (days)	Alignment
1	DP	F	68	19	135	40	110	85	30	78	130	6	2	119.2	7	Acceptable
2	MIPO	M	58	18	125	45	115	75	25	79	150	5	2	130.2	13	Acceptable
3	MIPO	M	55	17	140	45	110	80	25	88	145	5	1	125.4	12	Acceptable
4	MIPO	M	46	17	130	50	130	80	20	82	145	3	1	119.2	4	Acceptable
5	DP	F	75	17	125	35	95	85	20	81	120	7	1	125.8	10	Acceptable
6	MIPO	M	76	16	135	40	100	85	30	86	135	6	0	121.2	10	Acceptable
7	DP	F	40	16	130	40	105	80	30	80	140	6	1	111.3	6	Acceptable
8	DP	F	69	16	120	40	110	85	25	78	120	6	2	117.2	12	Acceptable
9	MIPO	F	83	15	115	35	110	75	30	75	145	5	0	116.5	3	Acceptable
10	MIPO	F	69	15	130	45	120	75	30	79	155	4	0	120.4	12	Acceptable
11	DP	M	52	15	140	40	115	70	25	76	125	8	3	114.8	6	Acceptable
12	DP	M	47	15	130	45	115	85	30	82	130	5	1	117.5	14	Acceptable
13	MIPO	M	54	14	120	45	110	85	25	78	150	6	1	119	8	Acceptable
14	DP	M	43	14	100	40	110	75	30	69	130	5	2	125.2	4	Acceptable
15	MIPO	M	81	13	120	45	115	70	30	77	145	4	3	123.6	6	Acceptable
16	DP	F	69	13	105	35	95	75	25	59	115	5	3	118	8	Acceptable
17	DP	F	43	13	110	40	100	85	30	72	130	7	0	119.8	7	Acceptable
18	DP	F	75	13	105	40	100	70	25	67	100	5	2	112	10	Acceptable
19	DP	F	76	12	105	35	95	70	30	67	120	5	2	122.4	6	Acceptable
20	DP	M	79	12	95	35	100	80	25	57	110	6	2	105.3	5	Unacceptable
21	DP	F	73	11	120	30	100	75	25	63	125	5	0	125.2	10	Acceptable
22	MIPO	F	74	9	100	40	95	80	25	55	145	3	1	120.1	8	Acceptable
23	DP	F	64	9	95	35	95	65	20	55	125	6	1	118.1	6	Acceptable
24	DP	M	68	8	95	35	95	75	30	43	125	6	1	106.2	7	Unacceptable
25	MIPO	F	62	7	110	45	100	70	30	53	140	4	0	124.1	12	Acceptable
26	MIPO	M	80	7	100	40	95	75	25	47	140	3	4	120.5	10	Acceptable
27	DP	M	84	7	95	25	110	70	20	43	110	7	1	108.2	11	Unacceptable
28	DP	F	64	7	95	35	90	70	25	41	125	7	2	119.2	7	Acceptable
29	DP	F	32	7	100	30	95	65	20	49	140	6	3	122.7	6	Acceptable
30	DP	F	73	7	85	20	100	65	20	46	110	8	3	125.2	10	Acceptable
31	DP	M	37	7	120	35	110	70	25	52	130	7	3	135.1	8	Acceptable
32	MIPO	F	60	7	115	30	95	75	25	53	130	4	2	131.3	8	Acceptable
33	MIPO	F	62	7	120	40	100	70	20	57	125	5	1	115.9	5	Acceptable

We found no significant differences in length of stay, range of motion in abduction, flexion, or internal/external rotation of the shoulder, constant score or VAS at the latest follow-up; however, the constant score in the MIPO group was slightly better (69.92 ± 14.51 vs. 62.90 ± 14.05). Flexion of elbow (142.31 ± 8.32 vs. 123.00 ± 10.18), posterior extension of shoulder (41.92 ± 5.22 vs. 35.50 ± 5.83), and postoperative VAS (4.38 ± 1.04 vs. 6.15 ± 0.99) were all significantly better in the MIPO group (*p* < 0.05) (Table 2).

**Table 2:** Statistical results

	MIPO	DP	*p* value
Age (y/o)	66.15 ± 11.83	61.55 ± 15.86	0.378
Gender (M/F)	7/6	7/13	0.284
Follow-up	12.46 ± 4.39	11.90 ± 3.85	0.320
Abduction (degrees)	120.00 ± 12.25	110.25 ± 16.02	0.072
Extension (degrees)	41.92 ± 5.22	35.50 ± 5.83	0.003^*^
Flexion (degrees)	107.31 ± 10.92	102.25 ± 7.69	0.128
Elbow flexion (degrees)	142.31 ± 8.32	123.00 ± 10.18	0.000*
External rotation	26.15 ± 3.63	25.50 ± 3.94	0.634
Internal rotation	76.54 ± 5.16	75.00 ± 7.26	0.513
Constant score	69.92 ± 14.51	62.90 ± 14.05	0.176
VAS post-op	4.38 ± 1.04	6.15 ± 0.99	0.000^*^
VAS latest F/U	1.23 ± 1.24	1.75 ± 0.97	0.186
Length of stay (days)	8.54 ± 3.31	8.00 ± 2.58	0.774
Head-shaft angle (degrees)	122.12 ± 4.70	118.42 ± 7.40	0.208
Alignment (acceptable/total)	13/13	17/20	0.143

*Significant difference is considered as *p* < 0.05

Malreduction was considered in three cases of the DP group (varus, 3 of 20), while the alignment of all cases in the MIPO group was found to be acceptable (0 of 13), though the difference was not significant (*p* = 0.143). No loss of reduction, screw cutout, or protrusion were found in the latest follow-up. Clinical and radiographic bone healing were considered in all cases. No signs of bone absorption or nonunion were discovered. Consolidation of fracture was observed in patients with more than 6 months of follow-up.

## Discussion

We conducted this single center retrospective study to introduce a modified approach for minimally invasive plating osteosynthesis of three-part proximal humeral fractures in comparison to the classic deltopectoral procedure.

In the 1970s, Neer described a four-segment classification system and treatment principles for the proximal humeral fracture, which has been widely used until now [8, 9]. Although the complication rate remains relatively high [3, 4, 6, 10, 11], surgical intervention, which may be technically demanding, should be considered for fractures with head-to-shaft displacement of over 50%, as well as varus or valgus deviation of over 20 degrees from the physiological value of 130° of head-to-shaft inclination [3, 9]. Various approaches could be selected based on the different types of fracture [12–17].

The classic deltopectoral approach is the most widely used approach for shoulder surgeries, especially for the surgical treatment of three-and four-part proximal humeral fractures [9]. This technique can provide effective visibility of the anterior and lateral aspects of the proximal humerus and can easily be extended proximally and distally if necessary. However, access to the posterior aspect of the shoulder and the exposure and fixation of a displaced greater tuberosity fragment may be difficult. Moreover, overzealous dissection of soft tissue may increase the risk of avascular necrosis of the humeral head [18].

Currently, the deltoid splitting approach is becoming a more attractive option in the treatment of two-or three-part proximal humeral fractures. This approach is the distal portion of a transacromial approach, which was first described by Kessel and Watson for the inspection and treatment of painful arc syndrome [19]. Different skin incisions may be selected for various purposes, including fracture treatment, arthroscopy, and arthroplasty [7, 12, 13, 15, 16, 19–26]. The deltoid splitting approach can provide good visibility of the posterolateral aspect of the proximal humerus, and the management of a displaced greater tuberosity can be achieved. With this approach, it also brings fewer disturbances to the medial soft tissue hinge, which is thought to decrease the risk of necrosis of the humeral head, although in some two- or three-part cases, dissection of the medial osteosteal hinge may be unnecessary. The anterolateral longitudinal incision approach can be used for minimally invasive procedures. Many studies have demonstrated good results for minimal invasive plate osteosynthesis (MIPO) in PHF, most of which are two-part cases [7, 15, 16, 21, 23–26]. Although rarely reported, it does put the axillary nerve at risk [5, 27, 28]. Injury to the axillary nerve is theoretically possible and has been supported by clinical and cadaveric studies [3–5, 27, 28]. Further, several anatomic studies have confirmed the location of the axillary nerve [27, 28]. Soft tissues by-passing the nerve should be kept at 5–7 cm at a minimum. Preoperative ultrasonography may help to identify the location of the zone at risk.

The MIPO approach is typically utilized in two-part fractures of the proximal humerus. It can provide good reduction and fixation, without concern of medial instability. However, in three-part cases, it may be difficult to restore stability to the medial head-shaft junction through a traditional MIPO approach. We have modified the MIPO procedure, with the belief that grasping the proximal fragment with locking screws and a reliable bone graft can provide enough stability to the whole structure, which was shown by clinical and radiographic evaluation at follow-ups.

Several studies have been conducted for the comparison of different approaches. Wu et al. analyzed traditional DP approach and the deltoid splitting approach in 63 patients, and no statistically significant differences were detected in clinical, radiographic, or electrophysiological outcomes [29]. In a prospective randomized study, Buecking et al. found no differences between the deltopectoral and deltoid splitting approaches in terms of complications, reoperations, and functional recovery [30]. However, none of the clinical studies have directly mentioned loss of range of motion due to adhesion and scar formation caused by dissecting the deltoid bursa in both approaches, which can be observed in part of the patients. Disturbance of the bicep tendon may also affect elbow flexion in some cases.

In the MIPO group in our series, dissection of the deltoid bursa was avoided by direct reduction inside the humeral head, traction of rotator cuff sutures, and indirect reduction of distal fragment. With this technique, the deltoid bursa should be kept untouched and intact. We have observed better range of motion in the mid-term follow-up, especially in terms of posterior extension, in comparison to the deltopectoral approach. Moreover, without disturbing the long head of the bicep, better flexion of the elbow can also be observed, although the evidence is limited. All steps of the procedure are performed at the periosteal level or in the humeral head. The axillary nerve is protected in the soft tissue of the lateral side, and the soft tissue hinge of the medial side remains intact, which we believe decreases the incidence of nerve involvement and avascular necrosis.

Studies have shown good results of intramedullary nailing for two- or three-part proximal fractures, and even in some cases of four-part fractures. Intramedullary nailing is a technically demanding procedure, with relatively longer study curve. It is important to find a correct entry point, as well as performing rotator cuff repair. The insertion of the nail may be blocked by temporary fixation of K-wires. Moreover, the nail itself may affect the operation of bone grafting on the medial side.

We have attempted to use this modified approach in some four-part fractures and found it to be difficult to pull the displaced lesser tuberosity back through the bony portal. It may be difficult to reduce the lesser tuberosity through a traditional approach as well, but the modified approach itself is not suitable for four-part fractures. In a systematic review, Gruson et al. concluded that isolated tuberosity fractures could be fixed arthroscopically [31]. With an additional arthroscopic procedure, fixation of the fourth part could be possible. The combination of arthroscopic and minimally invasive reduction and plating is shows potential for four-part fractures.

There are some limitations in this study. Specifically, it was a single-center retrospective study, with a relatively small sample size. The average follow-up was not long enough to give a comprehensive evaluation to the modified approach; in particular, we did not include functional recovery, effectiveness of the bone graft, maintenance of the reduction, and outcome of the consolidation. However, with a minimum of 7 months of follow-up, significant differences can be detected. Additional multicenter, prospective, controlled studies with longer follow-up times may be necessary for a more accurate assessment of this approach.

## Conclusion

The use of a modified anterolateral approach and intra-osseous portal is safe and effective for minimally invasive reduction and locked plating treatment of three-part proximal humeral fractures, with better functional results compared to the deltopectoral approach. Further research is necessary to determine its utility in more complicated cases, including four-part fractures. The combination with arthroscopic procedures may expand the utility of this approach.

## Abbreviations

CS: Constant score
DP: Deltopectoral approach
MIPO: Minimal invasive approach
ORIF: Open reduction with internal fixation
PHF: Proximal humeral fracture
PHILOS: Precontoured locking plate
VAS: Visual analogue scale
